# Comparison between thyroid stimulating immunoglobulin and TSH-receptor antibodies in the diagnosis and management of Graves’ disease

**DOI:** 10.3389/fendo.2024.1487490

**Published:** 2024-11-28

**Authors:** Peiwei Yao, Yunliang Xie, Yunlin Wang, Chunyan Liang, Bingwen Huang

**Affiliations:** Endocrinology Department of Foshan Second People’s Hospital, Foshan, China

**Keywords:** TRAb thyroid antibodies, thyroid-stimulating immunoglobulin, Graves’ disease, anti-hyperthyroidism drug therapy, duration of treatment

## Abstract

**Introduction:**

TSH-receptor antibodies (TRAb) directed against the TSH receptor (TSH-R) induce hyperthyroidism in patients with Graves′ disease (GD). TRAb detected by previous radioimmunoassay only reflects the presence of autoantibodies, but not the function of such antibodies. Thyroid stimulating immunoglobulins (TSI) is a relatively new method for assessing TSH-receptor antibodies function. The aim of this study was to investigate the role of TSI in the diagnosis and management of GD.

**Methods:**

Patients with newly diagnosed GD (n=140, age 38.00 ± 11.99 years, 106 women) received pharmacological therapy (methimazole) up to 18 months in the outpatient or inpatient department of the Second People’s Hospital of Foshan City from January 2013 to December 2018. GD was identified by clinical signs and symptoms and relevant laboratory tests. Blood samples for TSI and TRAb and other thyroidal biomarkers were obtained at baseline and at three times during the follow-up. All patients with GD were followed up for at least 5 years to see if the patient was cured or had relapsed.

**Results:**

TSI and TRAb in GD patients were significantly higher than those in the normal control group (*P*<0.001), and there was a strong correlation between TSI and TRAb in GD patients (*P*<0.001). After treatment, TSI and TRAb significantly decreased (*P*<0.05), TSI and TRAb in patients with disease course more than 2 years were significantly higher than those in patients with disease course less than 2 years (*P*<0.05), There was no statistically significant difference in TSI and TRAb at initial diagnosis between patients with a disease duration of more than 2 years and less than 5 years and those with a disease duration of more than 5 years (*P*>0.05); if the patients were still positive for TSI or TRAb antibodies at 5 years of follow-up after treatment with anti-hyperthyroidism medication, the patients were at a higher risk of relapse (*P*<0.001).

**Conclusion:**

The higher TSI at the initial diagnosis of GD, the longer duration of treatment with anti-hyperthyroid drugs and the higher risk of relapse. Compared with TRAb, serum TSI level is also important in the clinical diagnosis and prognosis of GD, but which one is superior to the other needs further study.

## Introduction

Graves’ disease (GD), also known as toxic diffuse goiter, is a common specific autoimmune disease with a genetic predisposition, and epidemiological studies have indicated that Graves’ disease accounts for about 83% of all hyperthyroidism, and its pathogenesis is closely related to thyrotropin receptor body (TRAb) (1). By simulating the role of TSH, TRAb combines with TSHR to cause excessive growth and functional enhancement of thyroid cells, leading to goiter, hyperfunction and GD. Therefore, TRAb can be detected in the vast majority of GD patients and is considered as an important marker for the diagnosis, treatment and prognosis of GD in clinical work. The 2018 European Thyroid Society Graves Guidelines for the Management of Hyperthyroidism and the 2016 American Thyroid Society Guidelines for the Diagnosis and Treatment of Hyperthyroidism and Other Causes of Thyroidism both recommend TRAb as a diagnostic marker for hyperthyroidism (2, 3). TRAb is a heterogeneous antibody that can be divided into TSAb, TSBAb, and neutralizing antibodies. Among them, only stimulating TRAb, namely thyroid stimulating immunoglobulin, is present Immunoglobulin (TSI) is believed to be responsible for inducing sustained signaling activity in the thyroid gland, and is most closely related to GD (4). To date, TSI has been detected by bioassays and immunoassays. Immunological methods are highly sensitive, but cannot distinguish between types of TRAb. The bioassay using cell culture has not been approved for clinical diagnosis because of its cumbersome operation and many influencing factors. Recently, Siemens has successfully developed an assay based on the IMMULITE 2000 platform for direct measurement of TSI. However, the clinical diagnostic value of this method for GD has yet to be confirmed due to its recent clinical application (5).Current treatment options for Graves’ disease include Anti-hyperthyroidism drug (ATD) therapy, radioactive iodine therapy (iodine-131 therapy) and surgery. In Europe and China, ATD therapy is usually the treatment of choice for patients with GD, while radioactive iodine therapy is the first-line treatment option in the U.S. Regardless of the therapeutic measure, it should be considered in a comprehensive manner, which can be based on the patient’s specific situation, the advantages and disadvantages of the treatment modality, and the willingness to treat.

We know that the conventional treatment of ATD is usually recommended for 1 1/2 to 2 years, and most patients can be alleviated or even cured, but nearly 30% of patients cannot stop taking the drug for a long time or some patients are prone to relapse. TRAb is also recommended as a good prognosis and drug withdrawal indicator during treatment. Compared with TRAb, whether TSI can play a greater role in the diagnosis, treatment and prognosis of GD remains to be further explored. However, TSI has not been widely used in some countries or regions, and it has not attracted sufficient attention from some clinicians. Therefore, the aim of this study was to compare whether there is a difference in the clinical significance of TSI and TRAb in the diagnosis and management of GD patients treated with anti-hyperthyroid drugs.

## Materials and methods

### Study subjects

A total of 140 GD patients (36 males and 104 females) who were newly diagnosed and followed up in the outpatient or inpatient department of the Second People’s Hospital of Foshan City from January 2013 to December 2018 were retrospectively analyzed and compiled as the observation group. The diagnostic criteria for GD are firstly a diagnosis of hyperthyroidism, followed by diffuse enlargement of the thyroid gland, and in a few cases no enlargement of the thyroid gland. Since TRAB and TSI can be detected in the vast majority of patients, many international guidelines have already recommended that these antibodies be a requirement for diagnosis. In clinical practice, TPOAb and TgAb are elevated in patients with GD, but the guidelines suggest that the diagnosis of GD should not be based on high titer serum levels of TPOAb and TgAb.

In addition, 24 healthy (5 males and 19 females) were treated as normal control groups. All patients were examined at the first visit, including demographic characteristics (sex and age), personal and family history records, and the diagnosis of GD was made based on medical history, physical examination, routine thyroid function and antibody tests (TSH, FT3, FT4, TSI, TRAb, TgAb, TPOAb), and thyroid ultrasound. Radioactive iodine therapy, withdrawal from drug regimens is also excluded, and patients who change or take propylthiouracil are not included). TSI and TRAb of GD patients were recorded at the first visit, 12 months and 24 months, and all patients were continuously followed up and evaluated whether they were cured or not according to their clinical symptoms and related biochemical indexes at the fifth year (if clinical symptoms recurred after drug withdrawal and abnormalities such as thyroid function were reviewed, relapse was considered).

### Assays

The healthcare workers used red-tipped dry tubes to draw the patients’ blood, which was sent to the testing department on the same day to separate the patients’ serum and then refrigerated at 2-8°C and tested within one week. and serum TSH, FT3, FT4 and TRAb levels were detected by Roche E602 electrochemical immunoluminescence instrument and its accompanying reagents, and TSI levels were detected by Siemens IMMULITE2000XPI automatic chemiluminescence immunoassay analyzer and its accompanying kit. Normal Reference Ranges TSH 0.350-4.940 μIU/mL, FT4 9.01-19.05 pmol/L, FT3 2.63-5.70 pmol/L, TRAb 0.00-1.75 IU/L, TSI 0.00-0.55 IU/L IMMULITE 2000XPI Fully Automated Chemiluminescent TSI Analyzer The laboratory analytical performance meets manufacturer’s claims.

### Statistical analysis

SPSS 21.0 software package was used for statistical analysis. The normally distributed measures were expressed as mean ± standard deviations (x ± s), and the non-normally distributed measures were expressed as Md (P25, P75). The independent-samples t-test or one-way ANOVA was used for the normally distributed continuous variables, and the nonparametric test was used for the non-normally distributed continuous variables. *P*<0.05 indicated a statistically significant difference, and *P*<0.01 indicated a significant difference. Correlation analysis of variables was performed using the spearman analysis method.

## Results

A total of 140 GD patients were collected and followed up, including 34 males and 106 females, with an average age of (38.00 ± 11.99) years and a median course of disease of 48 months. The median FT4, TRAb and TSI levels were 30.71 pmol/l, 10.44 IU/L and 9.18 IU/L, respectively (**Table 1**).

**Table 1:** Characteristics of the patients at the time of diagnosis [*x ± s*; *Md* (*P25, P75*)].

	GD	Healthy person	P value
n	140	24	
Age at presentation(years)	38.00 ± 11.99	43.58 ± 17.13	0.051
Female/male	34/106	5/19	0.801
course of disease, month	48 (24,69)		
FT4, pmol/l	30.71 (20.11,44.82)	12.31 (11.54,13.19)	<0.001
FT3, pmol/l	11.60 (5.16,28.05)	4.15 (3.57,4.57)	<0.001
TSH, mIU/L	0.004 (0.003,0.013)	1.881 (0.885,2.674)	<0.001
TRAb, IU/L	10.44 (6.47,22.98)	0.83 (0.80,1.03)	<0.001
TSI, IU/L	9.18 (5.97,18.75)	0.44 (0.20,0.69)	<0.001
TPOAB, IU/mL	590.71 (50.10,1000.00)	23.79 (1.06,134.47)	<0.001
TGAB, IU/mL	107.82 (8.11,499.40)	13.36 (1.72,76.32)	<0.01

TSI, thyroid-stimulating immunoglobulin; TRAb, thyrotropin receptor antibody; TSH, thyroid stimulating hormone; TPOAB, Thyroid Peroxidase Antibodies; TGAB, Thyroglobulin Antibodies; FT4, Free T4; FT3, Free T3.

**Figure 1:**
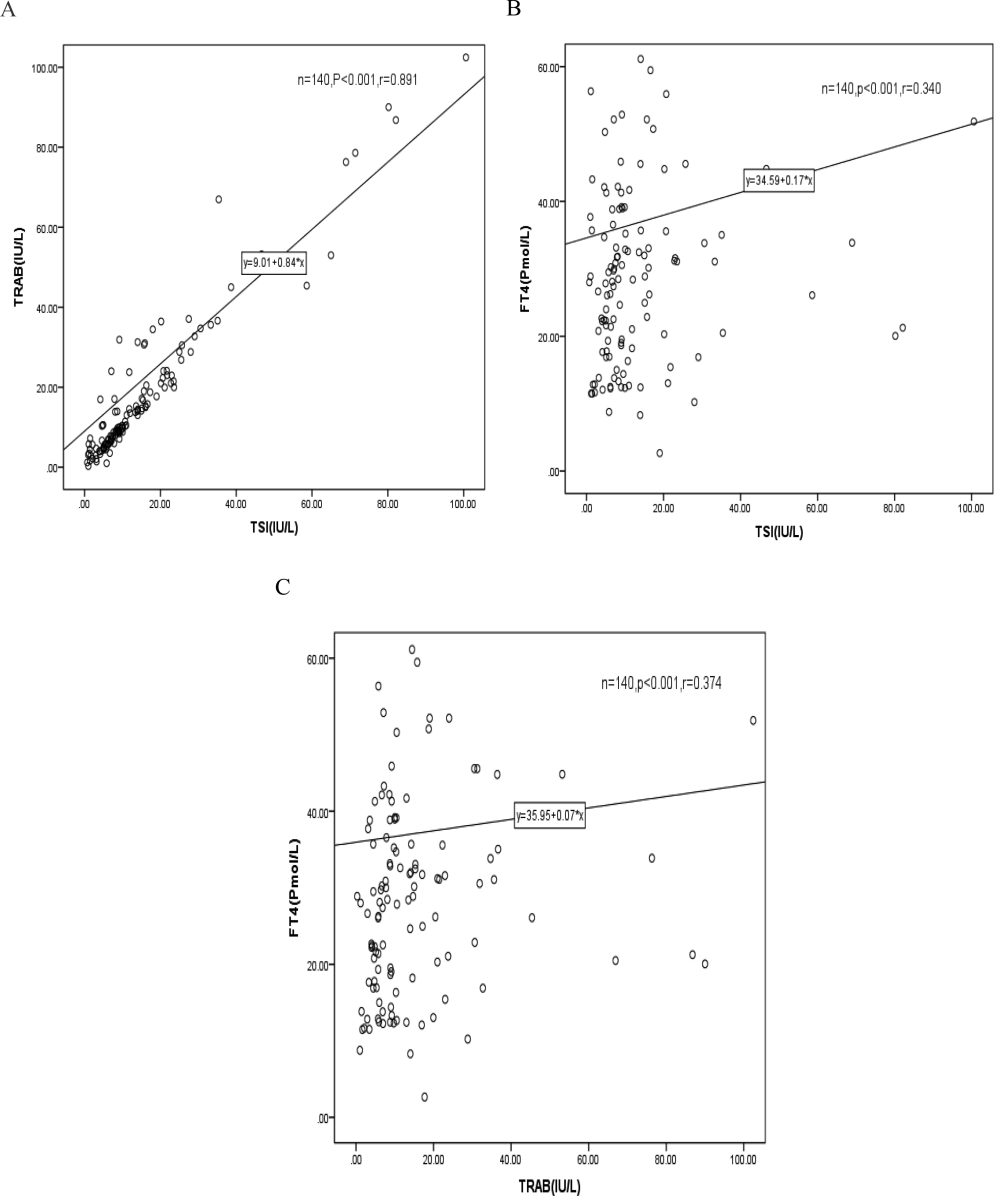
Correlation between TSI vs TRAb **(A)**, TSI vs fT4 **(B)** and TRAb vs fT4 **(C)** at initial diagnosis. r_s_ = Spearman’s Rho Coefficient.

For all subjects, TSI was highly correlated with TRAb at the time of diagnosis (*r_s_* =0.891, *p*<0.001, **Figure 1A**), TSI was correlated with FT4 at the time of diagnosis (*r_S_* =0.340, *p*<0.001, **Figure 1B**), TRAb was correlated with FT4 at the time of diagnosis (*r_s_* =0.374, *p*<0.001, **Figure 1C**).

All GD patients had significantly elevated TSI and TRAb levels at initial diagnosis. Compared with the initial diagnosis, levels of TSI and TRAb decreased significantly at 12months and 24 months, compared to 12 months, TSI is lower at 24 months, as is TRAb, and the difference was statistically significant (*p*< 0.05) (**Table 2**).

**Table 2:** TSI, TRAb at different times presented as median and range in GD.

	n	initial diagnosis	12 month	24 month	P value
TRAB (IU/L)	140	10.44 (6.47,22.98)	3.27 (1.75,6.14) *	1.67 (0.91,3.75) *†	< 0.05.
TSI (IU/L)	140	9.18 (5.97,18.75)	2.37 (0.97,5.00) *	1.35 (0.81,2.86) *†	< 0.05.

Compared with initial diagnosis, *P < 0.05; Compared with 12 months, †*P* <0.05.

We divided the GD patients into 3 groups according to their disease duration. TSI and TRAB at initial diagnosis were significantly lower in patients with a disease duration of 2 years than in patients with a disease duration of more than 2 years (*p*< 0.05). However, there was no statistically significant difference in TSI and TRAb levels at initial diagnosis between GD patients with a disease duration of 2 to 5 years and those with a disease duration of more than 5 years (*p*> 0.05) (**Table 3**). The lower the levels of TSI and TRAb at initial diagnosis, the shorter the disease duration seemed to be.

**Table 3:** TSI, TRAb at initial diagnosis presented as median and range in GD with different treatment duration.

Duration of treatment	n	TSI(IU/L)	TRAB(IU/L)
Within 2 years	46	7.62 (4.58,11.28)	8.68 (5.50,14.11)
2-5 years	58	10.00 (6.81,20.72) ||	12.45 (8.37,28.82) **
5+ years	36	14.50 (5.97,23.37) ¶*	16.29 (5.93,31.73) ††*

Compared with the disease course within 2 years, ||*P* 0.031 (*P*<0.05), ¶*P* < 0.028 (*P*<0.05); Compared with the disease course within 2 years, **P 0.033 (*P*<0.05) ††*P* < 0.040 (*P*<0.05); There was no statistically significant *P >0.05 compared to 2 to 5 years of disease.

Ninety-nine of 140 GD patients who were TSI-positive at initial diagnosis became TSI-negative after a standard course of medication, and only eight of these 99 patients relapsed at 5 years of follow-up. Nineteen patients remained TSI-positive during treatment, only three of these patients were able to discontinue the drug at 5 years of follow-up, and 16 patients had recurrent symptoms and were prone to relapse. Twenty-two patients with GD had complex changes in TSI during treatment, which could be high or low, sometimes negative, sometimes positive, and only 6 of these patients could be cured even after 5 years of follow-up, while 16 patients were prone to relapse and still needed to take medication. Lower recurrence rates in TSI- and TRAb -negative patients compared to patients who remain TSI- and TRAb -positive after drug treatment (*p*< 0.001) (**Table 4**).

**Table 4:** Changes of TSI and TRAb over 5years in 140 TSI-positive Graves’ patients with hyperthyroidism.

	Recovered(n=100)	Remission or recurrence (n=40)	p-value
TSI			0.001
Positive	3	16	
Negative	91	8	
Complex changes	6	16	
TRAB			0.001
Positive	7	21	
Negative	85	7	
Complex changes	8	12	

Complex changes: Patients mainly present with antibody-negative and then re-positive conditions during the course of treatment.

Since the majority of GD patients in the clinic can be detected with the presence of these two antibodies, TSI and TRAb are of great value in the diagnosis of GD. However, in practice, we still find that there are rare cases where only one of the two antibodies is positive. We’ve collected a few extra cases like this. It was found that such patients seemed to have low antibody levels and a short overall disease duration (**Table 5**).

**Table 5:** Characteristics of patients who were only positive for one of the two markers with hyperthyroidism.

	negative TRAb and positive TSI	negative TSI and positive TRAb
N	3	4
Age (years)	48.00 ± 3.1	49 ± 5.4
Female/male	3/0	3/1
TRAb, IU/L	1.33 ± 0.25	2.87 ± 0.36
TSI, IU/L	1.26 ± 0.28	0.39 ± 0.07
course of disease, month	19.00 ± 1.5	17 ± 2.3

## Discussion

According to the diagnostic criteria of GD, many authoritative guidelines and clinical studies believe that TRAb is a valid test index, and should be tested in combination with thyroxine, triiodothyronine and thyroid stimulating hormone (3). But we know that TRAb can only reflect the GD patient’s autoantibodies against thyroid hormone receptor, cannot reflect the function of the antibody, The main problem of TRAb detection is that patients with chronic thyroiditis with hypothyroidism may also have false positive results, and the reason may be related to the presence of neutral TRAb or inhibitory TRAb in these patients, or it is possible that the patients themselves have chronic thyroiditis with GD (6).

TRAb, as a heterogeneous antibody, can be classified into TSAb, TSBAb, and neutralizing antibodies. Among them, TSAB, also known as TSI, is considered to be the one that is closely associated with GD-related hyperthyroidism, and some findings suggest that TSI is also closely associated with the severity of the disease and extra-thyroidal manifestations, such as thyroid ophthalmopathy (7, 8). Therefore, some scholars have suggested that TSI should also be used in the same way as TRAb, not only for the screening of GD, but also for the evaluation of the treatment effect and prognosis of GD patients (9). However, some scholars believe that compared with TRAb, TSI does not show more clinical benefit in the prognosis and management of GO (10). In practice, the clinical role of TSI has not been widely emphasized, and in some regions, there is no relevant program to carry out testing. Our study found that high levels of TSI and TRAb were detected in almost all GD patients, and that the levels of TSI and TRAb in GD patients were related to thyroid function status. The results of correlation analysis showed that the levels of TSI and TRAb were strongly correlated, the levels of TSI and TRAb were significantly correlated with FT4 levels but weakly correlated, and. The correlation between TSI levels and FT4 was essentially the same as the correlation between TRAb levels and FT4, which is in general agreement with some scholars’ studies (11) Therefore, we believe that TSI can be used as one of the screening and diagnostic indicators for GD patients in China, and it is recommended to use it in combination with TRAb detection to improve the accuracy of diagnosis, and it is recommended to establish the corresponding reference interval for the level of medical determination of TSI in different regions to achieve the best clinical diagnostic performance of GD (12).

In terms of the evaluation of the treatment effect, Scappaticcio L et al. found a stronger correlation between TSI and FT4 than that between TRAb and FT4 (13). As a sensitive indicator of thyroid function status, the FT4 level decreased gradually with the disease remission after ATD treatment. Our study also found that, along with the remission of disease after ATD treatment, the level of TSI and TRAb antibody also decreased significantly. Therefore, we believe that TSI can also be used as a sensitive indicator to determine the ability of methimazole treatment in GD patients.

In terms of prognosis, we know that currently there are three main treatment methods for hyperthyroidism, namely drug therapy, surgery and radioactive iodine therapy, among which drug therapy is the first choice in most countries as the first-line treatment, while the traditional clinical experience recommended drug therapy course is 18-24 months, and TRAb is also considered to be a good indicator to predict drug withdrawal. However, in actual clinical work, we find it difficult to achieve this. Even if the patients have good compliance, take medicine on time, check regularly and do not have any bad habits, only 6-70% of patients can fully recover, and about 30% of patients have a long treatment time and a high risk of recurrence. In a study by Zhou YL et al., about 20% of TRAb-negative GD patients relapsed and most of them were TSI-positive, suggesting that discontinuing an ATD when both TSI and TRAb are negative significantly reduces the risk of relapse (14). Hwang S et al. found that the prediction of GD relapse by the level of TSI that can be detected has a higher specificity and predictive value (15). Several studies have shown that TSI is a good predictor of relapse in GD patients treated with ATD (16–18). In this study, we found that the higher the levels of TSI and TRAb at the initial diagnosis of GD patients, the longer the duration of the disease may be, and the length of drug treatment for these patients may be more than 1 ½ to 2 years if they take medication, and the risk of relapse is high, and in the course of the follow up of these patients, We found that patients with GD who were consistently positive for TSI antibodies or had complex changes were at higher risk of recurrence compared to patients who were negative for TSI antibodies whereas there was no statistically significant difference in the risk of recurrence between GD patients with persistently positive antibodies and complicated changes (*P* 0.466); GD patients with persistently positive TRAb antibodies or with complex changes had a higher risk of relapse than those with negative TRAb antibodies, and no statistically significant difference in the risk of relapse between persistently positive antibodies and complex changes (*P* 0.349). After a long course of treatment, the antibody level decreased significantly but still not completely normal, the probability of recurrence is high.

Although TSI does not show much superiority in prognosis compared with TRAb, we think that it can be considered to predict the possible course of treatment and recurrence risk of patients at the time of initial diagnosis by jointly detecting TSI and TRAb levels, so as to help patients choose the appropriate treatment plan better. When the TRAb of GD patients is negative and TSI is positive, it is still recommended to extend the treatment time of ATD appropriately.

It is very rare for GD patients to be positive for only one of the two markers. So it’s hard to analyze statistically whether it’s clinically significant or not. and we will continue to keep an eye out for such cases to expand our study more deeply. There are still shortcomings in this study. First, the sample size of this study is small and may lead to potential bias due to regional differences, and more regions as well as more GD patients should be included for analysis. Second, the follow-up period was not long enough to determine the prognostic value of TSI compared with TRAb in GD patients completely and accurately, a longer follow-up period is needed to validate the conclusions.

In conclusion, for patients with GD who were given a course of standard anti-hyperthyroid drugs, if the TSI is still positive, it is recommended to appropriately prolong the duration of ATD treatment to reduce the recurrence rate.

## Data Availability

The original contributions presented in the study are included in the article/supplementary material. Further inquiries can be directed to the corresponding author.

## References

[1] Smith TJ Hegedüs L. Graves' disease. N Engl J Med. (2016) 375:1552–65. doi: 10.1056/NEJMra1510030 27797318

[2] Kahaly GJ Bartalena L Hegedüs L Leenhardt L Poppe K Pearce SH. 2018 European thyroid association guideline for the management of graves' hyperthyroidism. Eur Thyroid J. (2018) 7:167–86. doi: 10.1159/000490384 PMC6140607 30283735

[3] Ross DS Burch HB Cooper DS Greenlee MC Laurberg P Maia AL. 2016 American thyroid association guidelines for diagnosis and management of hyperthyroidism and other causes of thyrotoxicosis. Thyroid. (2016) 26:1343–421. doi: 10.1089/thy.2016.0229 27521067

[4] Morshed SA Davies TF. Graves' disease mechanisms: the role of stimulating, blocking, and cleavage region TSH receptor antibodies. Horm Metab Res. (2015) 47:727–34. doi: 10.1055/s-0035-1559633 PMC5047290 26361259

[5] van Balkum M Schreurs MWJ Visser WE Peeters RP Dik WA. Comparison of two different TSH-receptor antibody assays: A clinical practice study. Heliyon. (2023) 9:e22468. doi: 10.1016/j.heliyon.2023.e22468 38107298 PMC10724564

[6] Takasu N Matsushita M. Changes of TSH-stimulation blocking antibody (TSBAb) and thyroid stimulating antibody (TSAb) over 10 years in 34 TSBAb-positive patients with hypothyroidism and in 98 TSAb-positive graves’ patients with hyperthyroidism: reevaluation of TSBAb and TSAb in TSH-receptor-antibody (TRAb)-positive patients. J Thyroid Res. (2012) 2012:1–11. doi: 10.1155/2012/182176 PMC3359712 22655217

[7] Kahaly GJ Diana T Kanitz M Frommer L Olivo PD. Prospective trial of functional thyrotropin receptor antibodies in Graves disease. J Clin Endocrinol Metab. (2019) 105:e1006–14. doi: 10.1210/clinem/dgz292 PMC7067543 31865369

[8] Diana T Ponto KA Kahaly GJ. Thyrotropin receptor antibodies and Graves' orbitopathy. J Endocrinol Invest. (2021) 44:703–12. doi: 10.1007/s40618-020-01380-9 PMC8310479 32749654

[9] Chen Y Tan J Li X Chen G Wang Z Xu S. Correlation between thyrotropin receptor antibody and thyroid-stimulating immunoglobulin and methimazole responsiveness in patients with Graves' disease. J Chongqing Med Univ. (2022) 47:1327–32. doi: 10.13406/j.cnki.cyxb.003132

[10] Khamisi S Lundqvist M Engström BE Larsson A Karlsson FA Ljunggren Ö. Comparison between thyroid stimulating immunoglobulin and TSH-receptor antibodies in management of Graves ‘orbitopathy. Exp Clin Endocrinol Diabetes. (2023) 131(4):236–41. doi: 10.1055/a-2021-0596 PMC10158629 36706788

[11] Liu K Fu Y Li T Liu S Chen D Zhao C. Clinical efficacy of thyroid-stimulating immunoglobulin detection for diagnosing Graves' disease and predictors of responsiveness to methimazole. Clin Biochem. (2021) 97:34–40. doi: 10.1016/j.clinbiochem.2021.07.014 34331946

[12] Cheng X Chai X Ma C Jia Q Zhao H Dong Z. Clinical diagnostic performance of a fully automated TSI immunoassay vs. that of an automated anti-TSHR immunoassay for Graves*'* disease: a Chinese multicenter study. Endocrine. (2021) 71:139–48. doi: 10.1007/s12020-020-02386-2 32562184

[13] Scappaticcio L Trimboli P Keller F Imperiali M Piccardo A Giovanella L. Diagnostic testing for Graves' or non-Graves' hyperthyroidism: a comparison of two thyrotropin receptor antibody immunoassays with thyroid scintigraphy and ultrasonography. Clin Endocrinol (Oxf). (2020) 92:169–78. doi: 10.1111/cen.14130 31742747

[14] Zhou Y Zhou M Qi Y Wang W Chen X Wang S. The prognostic value of thyroid-stimulating immunoglobulin in the management of Graves' disease. Ther Adv Endocrinol Metab. (2021) 12:20420188211044943. doi: 10.1177/20420188211044943 34603682 PMC8481717

[15] Hwang S Shin DY Song MK Lee EJ. High cut-off value of a chimeric TSH receptor (Mc4)-based bioassay may improve prediction of relapse in Graves' disease for 12 months. Endocrine. (2015) 48:89–95. doi: 10.1007/s12020-014-0325-8 24968734

[16] Da Silva Santos T Oliveira JC Freitas C Couto de Carvalho A. Thyroid-stimulatory antibody as a predictive factor for Graves' disease relapse. Cureus. (2022) 14:e22190. doi: 10.7759/cureus.22190 35178331 PMC8843073

[17] Kwon H Kim WG Jang EK Kim M Park S Jeon MJ. Usefulness of measuring thyroid stimulating antibody at the time of antithyroid drug withdrawal for predicting relapse of Graves’ disease. Endocrinol Metab (Seoul). (2016) 31:300–10. doi: 10.3803/EnM.2016.31.2.300 PMC4923415 27118279

[18] Baek HS Lee J Jeong CH Lee J Ha J Jo K. The prediction model using thyroid-stimulating immunoglobulin bioassay for relapse of Graves' disease. J Endocr Soc. (2022) 6:bvac023. doi: 10.1210/jendso/bvac023 35441120 PMC9012332

